# Cell Shape and Surface Colonisation in the Diatom Genus *Cocconeis*—An Opportunity to Explore Bio-Inspired Shape Packing?

**DOI:** 10.3390/biomimetics4020029

**Published:** 2019-03-31

**Authors:** Timothy Sullivan

**Affiliations:** School of Biological, Earth and Environmental Sciences, Distillery Fields, North Mall Campus, University College Cork, T23 N73K Cork, Ireland; timothy.sullivan@ucc.ie; Tel.: +353-21-490-4662

**Keywords:** diatoms, biofilms, interfaces, ellipse, optimal packing, biomimetics

## Abstract

Optimal packing of 2 and 3-D shapes in confined spaces has long been of practical and theoretical interest, particularly as it has been discovered that rotatable ellipses (or ellipsoids in the 3-D case) can, for example, have higher packing densities than disks (or spheres in the 3-D case). Benthic diatoms, particularly those of the genus *Cocconeis* (Ehr.)—which are widely regarded as prolific colonisers of immersed surfaces—often have a flattened (adnate) cell shape and an approximately elliptical outline or “footprint” that allows them to closely contact the substratum. Adoption of this shape may give these cells a number of advantages as they colonise surfaces, such as a higher packing fraction for colonies on a surface for more efficient use of limited space, or an increased contact between individual cells when cell abundances are high, enabling the cells to minimize energy use and maximize packing (and biofilm) stability on a surface. Here, the outline shapes of individual diatom cells are measured using scanning electron and epifluorescence microscopy to discover if the average cell shape compares favourably with those predicted by theoretical modelling of efficient 2-D ellipse packing. It is found that the aspect ratio of measured cells in close association in a biofilm—which are broadly elliptical in shape—do indeed fall within the range theoretically predicted for optimal packing, but that the shape of individual diatoms also differ subtly from that of a true ellipse. The significance of these differences for optimal packing of 2-D shapes on surfaces is not understood at present, but may represent an opportunity to further explore bio-inspired design shapes for the optimal packing of shapes on surfaces.

## 1. Introduction

Benthic diatoms are a group of unicellular algae that are globally widespread in aquatic systems, and that are well known for their highly silicified and ornamented outer cell casing or frustule [1]. This group demonstrate remarkably varied cell shapes and adaptations to life at interfaces [2], and are often early and successful colonisers of both artificial and natural illuminated immersed substrates [3]. Aside from intrinsic interest in diatoms, understanding key factors that influence benthic diatom colonisation of substrates is also of importance in the development of novel effective antifouling technologies, particularly where such technologies incorporate control of surface roughness or engineered surface topography to disrupt cell settlement patterns [4,5]. 

Monoraphid diatom species of the genus *Cocconeis* (Bacillariophyta) are widespread (with some 812 species and intraspecific taxa of *Cocconeis* having been published to date, although several have not been validated [6]), and appear highly adapted to a lifecycle attached to a variety of surfaces, including whale skin [7], marine algae [8], bivalves, and crustaceans, among others [9,10]. The adaptations that control surface adhesion and biosilicification, and that lead to successful colonisation and proliferation on surfaces amongst this genus, and others, are therefore of great interest from a biomimetic or bio-inspired perspective [11,12,13,14], especially where the development of novel foul-release antifouling materials are concerned, for example [3]. 

Some benthic diatom species, particularly in the genus *Cocconeis*, are reported to form closely packed, near monospecies colonies on surfaces. For example, Molino et al. [12] have proposed a model of marine benthic diatom colonisation of submerged surfaces that involves packing of these *Cocconeis* cells in a monolayer on the surface in question. The 2-D cell outline (or individual valve shape) of these marine diatoms in contact with a substrate is also reported as elliptical in shape, or broadly elliptic with rounded apices [8,12,15,16], and it is known that the packing fraction (or packing density (φ)) achieved with 2-D elliptical shapes is crucially dependent upon aspect ratio (α) and upper and lower dimensional bounds of a given set of ellipses (see, for example, [17]). Therefore, questions arise as to whether the frustule shape and aspect ratio of these diatom cells fall within the theoretical range required for optimum shape packing, and indeed whether the adoption of this shape can confer any specific advantage for surface colonisation and space usage amongst those diatom species that display an (approximately) elliptical frustule.

To attempt to answer these questions, it is necessary to establish the true shape and aspect ratio range of these diatom cells. As such, these rigid highly silicified elliptical diatom cells may be treated as quasi-2D non-overlapping polydisperse (broadly) elliptical particles that can be packed on a surface (see, for example, [18]). In addition, due to the mechanisms of cell division among diatoms, these elliptical cells have both an upper and lower bound in the absolute dimensions that can be encountered in individual species. This is significant in terms of random shape packing, as it allows direct comparisons between predicted theoretical 2-D modelling of ellipse packing and those shapes and dimensions adopted by nature.

In this work, biofilm formation and surface coverage of diatom cells broadly identified as belonging to the genus *Cocconeis* are examined. The aspect ratios (major to minor axes lengths) of individual diatom frustules within these biofilms are measured using electron micrographs and compared with automated measurements using epifluorescence images of diatom colonies. These measurements are compared with the results from literature values derived using theoretical models of the aspect ratio necessary for optimal packing of 2-D ellipses [17,18].

## 2. Materials and Methods

Clean glass microscope slides (75 × 25 × 1 mm^3^, Corning) were submerged for between 5 and 30 days at approximately 1 m depth in July/August 2011 on the south coast of Ireland (51°29′39.1″ N 9°17′41.8″ W) to allow diatom colonisation. Slides were removed at intervals and carefully transferred to 50 mL Falcon tubes filled with artificial seawater (Tropic Marine, Wartenberg, Germany) containing 2.5% (*v*/*v*) glutaraldehyde (Sigma Aldrich, Dublin, Ireland) before transportation to the laboratory for further examination. In addition, marine biofilms formed on the peristracum of blue mussel, *Mytilus edulis*, were examined to confirm *Cocconeis* colonisation on natural surfaces compared to smooth glass substrates at the study location. 

### 2.1. Scanning Electron Microscopy of Diatom-Colonised Surfaces

Substrates containing diatom cells were divided into 1 cm^2^ sections and gently rinsed in fresh water to remove any salts present, before drying at room temperature for 48–72 h. These samples were subsequently coated with gold (layer thickness approximately 30 nm) before imaging by SEM (Hitachi S3400-N, Tokyo, Japan). Near-elliptical diatom frustules or valves of species that were broadly identified using ID features (for example, detail of external striation, internal raphe endings where possible, valvocopula, etc.) described by De Stefano et al., and others, [8,15,19] or Round et al., [1] as largely belonging to *Cocconeis* genus (for example, *C. scutellum* var. posidoniae; *C. scutellum* var. scutellum, *C. neothumensis* var. marina, or *C. stauroneiformis*). Note that not all individual near-elliptical diatom cells that were measured in this study were conclusively identified to species level, however, all measured cells were near-elliptical in outline and were broadly confirmed, where possible, as members of the genus *Cocconeis*. Images of individual valves and frustules were subsequently imported into ImageJ analysis software (Version 1.51a) and measured by overlaying an ellipse with major and minor axes lengths matching the maximum length and width of the selected frustule or valve.

### 2.2. Epifluorescence Microscopy

To compare and confirm SEM analysis in case of vacuum artefacts, replicate samples containing diatom-dominated biofilm were also immersed in an acridine orange solution (0.1% (*v*/*v*) in de-ionised water) for 2 min, and gently rinsed with freshwater, and imaged with an epifluorescence microscope (Leica, filter cubes H3 and I3, N Plan 40×/0.65) with a mercury bulb source. Dilute solution of acridine orange applied in this manner were found to stain the frustules of attached cells, meaning that intact frustules could be imaged and analysed on the glass substrates. These frustules were then measured using a semi-automated method as described below.

### 2.3. Data Acquisition and Ellipse Fitting

Data on frustule dimensions, including apical and transapical dimensions (otherwise referred to as major (*a*) and minor (*b*) axes lengths), aspect ratio ($\frac{a}{b}$), cell packing density, orientation, and surface area of fitted ellipses were acquired and calculated using ImageJ [20]. Images were imported and dimensions added using the “Set Scale” function and measured by overlaying an elliptical region coinciding with the minor and major axes lengths of the frustule of interest for manual acquisition from individual electron micrographs. Automated ellipse fitting was performed over larger areas using the inbuilt elliptical fitting tool and data collection features of ImageJ, using the same area, orientation, and centroid as the original selection. Statistical analyses were performed using the base package of R statistical software version 3.2 [21].

## 3. Results

**Figure 1:**
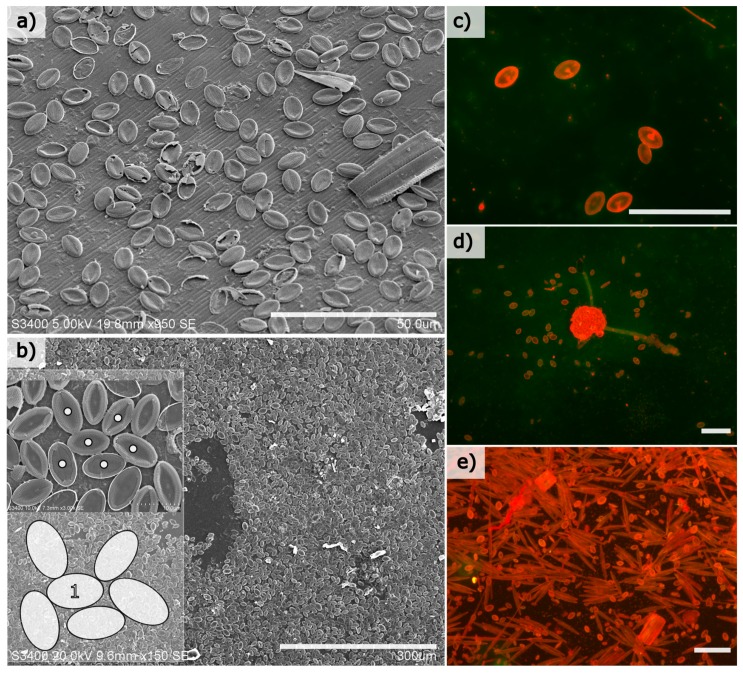
Representative scanning electron micrographs of elliptically shaped diatom frustules of various adnate diatom species (largely of the genus *Cocconeis*) attached to a natural surface such as the textured periostracum of *Mytilus edulis* (**a**) and smooth (at the macroscale) glass substrate (**b**) (scale bars = 50 and 300 µm, respectively). An example of the packing density of individual diatom cells is shown in the inset of (**b**), where individual cells are labelled with dots, and how individual cells can become “jammed” between surrounding cells at high abundance on a surface is illustrated in the cartoon inset, where the cell labelled “1” is prevented from rotating or moving by the surrounding cells. Epifluorescence images (**c**–**e**) show how cells initially colonise and distribute on a glass surface at low abundance after approximately 5–10 days immersion (**c**), until cells begin to proliferate on surfaces after approximately 14 days (**d**) until eventually forming the understory of an established and diverse diatom biofilm after 1 month (**e**) (each scale bar = 100 µm). Note that many of the other cell types in (**e**) are loosely attached to the glass surface and often have attachment strategies that differ from the adnate, near-elliptical-shaped cells that closely attach to the substrate.

Benthic diatoms colonised both natural and artificial surfaces at the experimental site within weeks of immersion, and the most prolific colonisers were members of the genus *Cocconeis* with an elliptical frustule shape. Cell abundances among this genus were calculated to exceed 10,000 cells/mm^2^ of substrate in mature biofilms (immersion period of 30 days in July/August 2011) (Figure 1b). Cells were generally restricted to a monolayer on both the glass and natural surfaces examined (Figure 1), and mature monolayers consist of both intact and damaged frustules, where it was observed that any space created by damaged cells was frequently re-colonised by smaller cells (not shown). 

**Figure 2:**
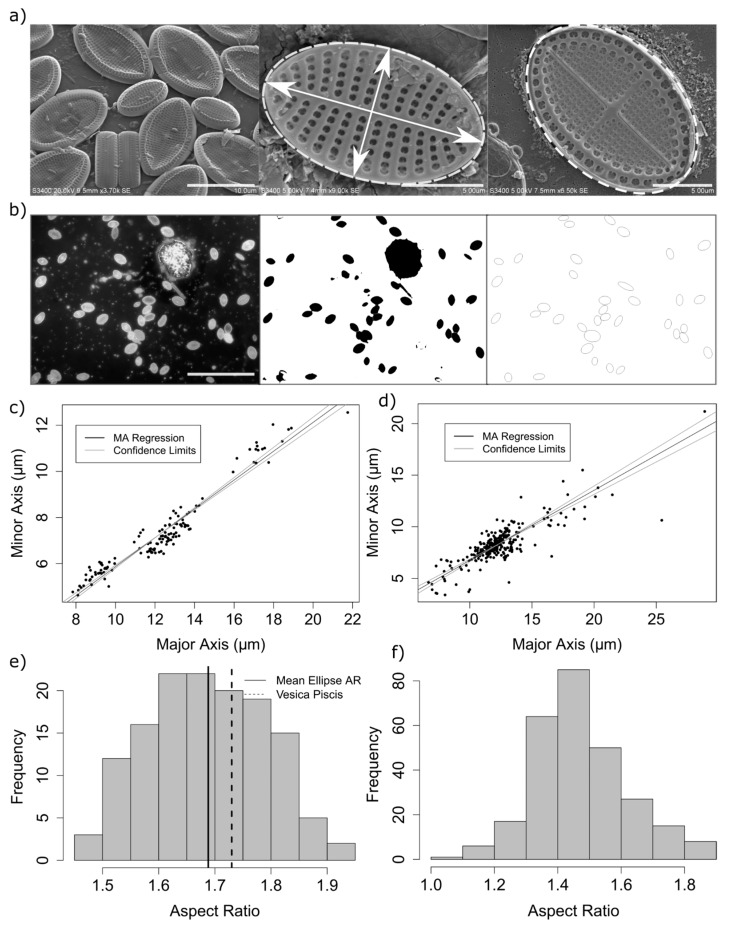
Measurement of aspect ratio (AR: minor axis/major axis) by both manual SEM measurement (**a**), showing a typical micrograph (left), measurement of an isolated frustule (middle) and the adhered partially intact lower valve (hypotheca) of a frustule (right). The process of automated epifluorescence measurements is shown (**b**), where staining with acridine orange (left) results in an epifluorescence image (left), thresholded (middle) and automated ellipse fitting to the resulting “particles” (right) allows more frustules to be measured. Minor axis linear regression (since errors can occur in both x and y variables) of 140 manually measured frustules from study location, showing a mean AR of 1.68 and a standard deviation of ±0.1 (**c**) and of automated measurements from 363 frustules using automated ellipse fitting to frustules isolated from epifluorescence image with a mean of 1.47 and a standard deviation ±0.2 (**d**). The distribution of the aspect ratios is shown for manually measured frustules using electron microscopy and a vesica piscis shape for comparison (**e**) and automated epifluorescence microscopy (**f**).

The aspect ratio of measured diatoms ranged from 1.45 to 1.9 (Figure 2), with a mean elliptical area in contact with the substrate of 73.3 µm^2^ (standard deviation = 35.7, *n* = 136). A small but significant difference was found between the mean aspect ratio as calculated by epifluorescence and electron microscopy measurement methods (mean aspect ratio using automated method = 1.47, mean using manual methods = 1.69, 95% confidence interval of the difference: 0.19–0.24, df = 360, with a Welch 2-sample *t*-test) (Figure 2). This difference is likely due to errors in the automated measurement method, including errors in the isolation and fitting of ellipses to selected regions of interest in the analysis of epifluorescence images whereby adjoining frustules were incorrectly measured, cells which were not of the genus *Cocconeis* were measured, or ellipses were erroneously fitted to surface debris. Therefore, it was found that careful manual isolation and measurement of cells from electron microscopy images, although from a smaller sample size, are therefore likely to better represent the true aspect ratio.

An aspect ratio between 1.5 and 1.8 is significant from the perspective of theoretical studies of random ellipse packing, as in this range, the packing density, jamming fraction, and number of contact points resulting from modelling of hard elliptical disks was found to vary with α [22]. For example, the average contact number and jamming density for bi-dispersed packed ellipses was found to reach a plateau above α = 1.5 [18]. Measurements of apical (major axis) length and transapical (minor axis) lengths from 140 cells from diatom monolayers consisting largely of *Cocconeis scutellum* var. posidoniae; *Cocconeis scutellum* var. scutellum or *Cocconeis neothumensis* var. marina, or *Cocconeis stauroneiformis* confirmed a linear relationship between minor and major elliptical axes of measured diatom cells ($r^{2}=0.93$) (Figure 2c,d).

## 4. Discussion

Intense competition can exist for available habitats among benthic organisms and, hence, suitable “new” surfaces immersed in aquatic environments are often rapidly colonised by competing organisms [23]. Diatoms, as photosynthetic unicellular organisms, are often regarded as being among the first colonisers of freshly immersed surfaces in aquatic environments [3]. As such, this group demonstrates specialised adaptations to life at surfaces and interfaces [24] and is a potentially rich source of bio-inspired design and biomimetic applications. These can include mechanisms of chemical communication [25], production of specialised extracellular polymeric substances for adhesion [26], mobility and surface exploration [27], and structural adaptations for enhanced resistance to mechanical damage [28]. Frustule morphologies and size ranges vary greatly among benthic diatoms, and these variations may be adaptations to different ecological niches [29]. For example, adnate cell forms may be preferable for avoiding predation or early colonisation of a surface, while stalks may have developed in some species to elevate cells above a substrate, providing possible advantages in photosynthesis and growth rates, for example. 

Molino et al. [12], have previously described a lifecycle strategy for *Cocconeis*-like diatoms that appears well-suited to colonisation of immersed surfaces (including marine coatings). The model of benthic diatom settlement and proliferation on a surface proposed by Molino et al. involves a process whereby cells demonstrate an initial period of motility on the substratum, gliding until they eventually “select” a position to permanently adhere. This permanently adhered cell, now referred to as a diatom “mother” cell, undergoes successive cell divisions, producing daughter cells where one produced cell remains in position while the other glides away to further explore the available surface, before themselves becoming mother cells. Molino et al. report up to four such cell divisions per day. This illustrates why some species in the genus *Cocconeis* are prolific colonisers of immersed surfaces, but also illustrates that optimal use of substrate within close proximity to the mother cell may be of key importance.

Adoption of a flattened elliptical frustule shape (or at least, broadly elliptical shape) or “footprint” may be a previously unrecognised aspect of enhanced surface colonisation capability. Theoretical modelling of polydisperse circular disks has demonstrated that these shapes pack randomly with a maximum packing density close to 0.84 [18] but, as an elliptical shape develops, the packing efficiency increases from an initial value of 0.837 to a maximum value of 0.895 for an aspect ratio, ∝ ≈ 1.43 (for =b/a). Other theoretical calculations of a 2-D elliptical shape, where overlapping and shape deformation are disallowed, indicate that a maximum packing fraction is reached at an aspect ratio of approximately 1.5 (for 10,000 particles) [30]. Therefore, approximately elliptical cells, like *Cocconeis*, could theoretically reach almost 90% coverage of a given surface area (excluding coverage by exopolymeric secretions). However, from a purely geometric perspective, adoption of an elliptical shape over a circular shape would represent an ≈6% increase in cell packing density on a surface at very high cell abundances. If disk rotations are disallowed then no improvement in packing fraction would occur [18], i.e., the additional rotational degree of freedom of an elliptical shape is essential for an increase in the packing fraction. This may suggest that *Cocconeis* cells may have also evolved an elliptical shape with an aspect ratio of this nature to facilitate mobility on a surface as the cell population increases rather than—or perhaps in addition to—optimised cell packing. At high cell abundances, where these species can utilise most of the available substrate area, an elliptical shape may therefore also aid in forming a cohesive biofilm as the number of contact points between cells increase, and the distance to neighbouring cells decreases. 

### Measured Deviations from an Elliptical Valve Outline 

**Figure 3:**
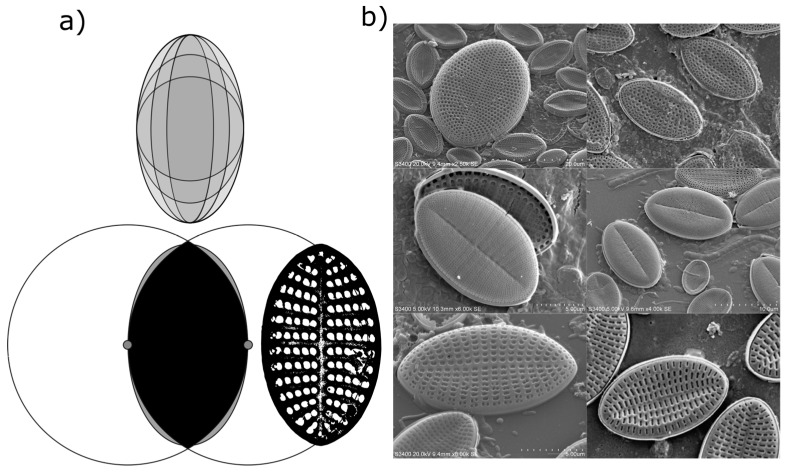
Examples of aspect ratios that a given ellipse might take ((**a**), top) when fitted to a frustule. The shape of many examined frustules was between that of an ellipse and a vesica piscis ((**a**), bottom), while (**b**) illustrates the variety of shapes encountered in surface-attached diatom frustules observed in the present study.

While valve shape of diatom species in the genus *Cocconeis* are often reported as near-elliptical, it is recognised that the true shape can vary from elliptic to broadly elliptic with rounded apices, or to slightly rhombic, for example [6]. While electron microscopy demonstrated that an ellipse is often a close fit to the outline of the diatom frustules in the current study, careful measurement of individual frustules also confirmed that the outline of individual frustules (or their component valves) was often not a true ellipse (Figure 3). Indeed, the outline of some measured frustules might be better described as intermediate between that of an ellipse and that of a vesica piscis (a shape that is the intersection of two disks with the same radius, intersecting in such a way that the centre of each disk lies on the perimeter of the other). A vesica piscis has an aspect ratio of 1.73, very close to that of the mean aspect ratio of the frustules measured manually in this study. Below an aspect ratio of 1.57, the surface area of a vesica piscis becomes larger than that of an ellipse of equivalent minor axis length, while at an aspect ratio of √3, and ellipse has an approximately 10% increase in area over that of the vesica piscis. The significance of this is not known from a space packing or cell motility perspective as yet, but may well be worth further exploration in terms of efficient 2-D modelling of the optimal shape of particles. 

The optimum conditions for individual diatom cell surface attachment, survival, and proliferation are, of course, very likely to be a function of the interaction of many complex variables, including available light, predation, the surface area available for adhesion (and the rugosity of the chosen substrate), the height profile and motility of the settling cell, and the proximity of nearby cells, for example. Determining the contribution and individual importance of any one of these factors is likely to be difficult to ascertain for the success of an individual colonising cell in different environmental conditions. However, we have shown here that *Cocconeis* cells, which are indeed regarded as prolific colonisers of specific substrates, appear to have a near-elliptical “footprint” in contact with the substratum, the aspect ratio of which appears to satisfy theoretical prediction for optimal random packing of hard elliptical shapes. 

This has potential applications in biomimetic design and in practical applications, such as the development of effective non-biocidal antifouling coatings against marine benthic diatom, for example, where an improved understanding of the dynamics of diatom settlement and adhesion is required, or could be further studied as, perhaps, a natural example of optimum use of space.

## 5. Conclusions

Aspect ratios of elliptical or near-elliptical diatom cells of between 1.48 and 1.93 were measured in this work, largely for the diatom genus *Cocconeis*. While the measured aspect ratios indicate that frustules of the genus *Cocconeis* often fall within those aspect ratios indicated by theoretical modelling for optimal hard 2-D elliptical particle packing, any improvement in packing density would be marginal (ca. 6% greater space utilisation over circles, for example). However, an elliptical shape may also facilitate the movement of individual cells (i.e., prevent cells from becoming restricted in movement between already settled cells as they glide across a surface, for example) at low cell abundances, but as a surface becomes more densely populated, the number of contact points between nearby cells at high cell abundances could be increased, perhaps providing greater biofilm stability. Subtle deviations from an elliptical shape in the outline of some diatom frustules were also measured. The theoretical and practical significance of these deviations should be explored from a biomimetic perspective in terms of optimal shape packing on surfaces, and also in overall terms, such as in understanding how such cell shapes influence surface colonisation success (and whether this information can be utilised, for example, for practical bio-inspired surface design for antifouling purposes).

## References

[1] Round F.E., Crawford R.M., Mann D.G. (1992). The Diatom. Biology & Morfology of the Genera.

[2] Cooksey K.E., Wigglesworth-Cooksey B. (1995). Adhesion of bacteria and diatoms to surfaces in the sea: A review. Aquat. Microb. Ecol..

[3] Zargiel K.A., Swain G.W. (2014). Static vs dynamic settlement and adhesion of diatoms to ship hull coatings. Biofouling.

[4] Sweat L.H., Johnson K.B. (2013). The effects of fine-scale substratum roughness on diatom community structure in estuarine biofilms. Biofouling.

[5] Scardino A.J., Guenther J., de Nys R. (2008). Attachment point theory revisited: The fouling response to a microtextured matrix. Biofouling.

[6] Al-Handal A.Y., Riaux-Gobin C., Jahn R., Wulff A.K., Minerovic A. (2019). Two new marine species of Cocconeis (Bacillariophyceae) from the west coast of Sweden. Eur. J. Taxon..

[7] Holmes R.W. (1985). The morphology of diatoms epizoic on cetaceans and their transfer from Cocconeis to two new genera, Bennettella and Epipellis. Br. Phycol. J..

[8] De Stefano M., Marino D., Mazzella L. (2000). Marine taxa of *Cocconeis* on leaves of *Posidonia oceanica*, including a new species and two new varieties. Eur. J. Phycol..

[9] Sullivan T., Regan F. (2017). Marine diatom settlement on microtextured materials in static field trials. J. Mater. Sci..

[10] Sullivan T., McGuinness K., Connor N.E.O., Regan F. (2014). Characterization and anti-settlement aspects of surface micro-structures from *Cancer pagurus*. Bioinspir. Biomim..

[11] Molino P.J., Hodson O.M., Quinn J.F., Wetherbee R. (2008). The Quartz Crystal Microbalance: A New Tool for the Investigation of the Bioadhesion of Diatoms to Surfaces of Differing Surface Energies. Langmuir.

[12] Molino P.J., Campbell E., Wetherbee R. (2009). Development of the initial diatom microfouling layer on antifouling and fouling-release surfaces in temperate and tropical Australia. Biofouling.

[13] Ragni R., Cicco S.R., Vona D., Farinola G.M. (2018). Multiple Routes to Smart Nanostructured Materials from Diatom Microalgae: A Chemical Perspective. Adv. Mater..

[14] Terracciano M., De Stefano L., Rea I. (2018). Diatoms Green Nanotechnology for Biosilica-Based Drug Delivery Systems. Pharmaceutics.

[15] De Stefano M., Romero O.E., Totti C. (2008). A comparative study of Cocconeis scutellum Ehrenberg and its varieties (Bacillariophyta). Bot. Mar..

[16] Romero O.E., Riaux-Gobin C. (2014). Two closely-related species of Cocconeis (Bacillariophyta): Comparative study and typification. Plant Ecol. Evol..

[17] Xu W., Chen H., Lv Z. (2010). A 2D elliptical model of random packing for aggregates in concrete. J. Wuhan Univ. Technol. Mater. Sci. Ed..

[18] Delaney G., Weaire D., Hutzler S., Murphy S. (2005). Random packing of elliptical disks. Philos. Mag. Lett..

[19] Majewska R., D’Alelio D., De Stefano M. (2014). Cocconeis Ehrenberg (Bacillariophyta), a genus dominating diatom communities associated with *Posidonia oceanica* Delile (monocotyledons) in the Mediterranean Sea. Aquat. Bot..

[20] Schneider C.A., Rasband W.S., Eliceiri K.W. (2012). NIH Image to ImageJ: 25 years of image analysis. Nat. Methods.

[21] R Core Team R (2016). A Language and Environment for Statistical Computing.

[22] Donev A., Cisse I., Sachs D., Variano E.A., Stillinger F.H., Connelly R., Torquato S., Chaikin P.M. (2004). Improving the density of jammed disordered packings using ellipsoids. Science.

[23] Wahl M. (1989). Marine epibiosis. I. Fouling and antifouling: Some basic aspects. Mar. Ecol. Prog. Ser.

[24] Wetherbee R., Lind J.L., Burke J., Quatrano R.S. (1998). Minireview—The first kiss: Establishment and control of initial adhesion by raphid diatoms. J. Phycol..

[25] Allen A.E., Vardi A., Bowler C. (2006). An ecological and evolutionary context for integrated nitrogen metabolism and related signaling pathways in marine diatoms. Curr. Opin. Plant Boil..

[26] Hoagland K.D., Rosowski J.R., Gretz M.R., Roemer S.C. (1993). Diatom Extracellular Polymeric Substances: Function, Fine Structure, Chemistry, and Physiology. J. Phycol..

[27] Consalvey M., Paterson M.D., Underwood G.J.C. (2004). The Ups and Downs of Life in a Benthic Biofilm: Migration of Benthic Diatoms. Diatom Res..

[28] Gebeshuber I.C., Kindt J.H., Thompson J.B., Del Amo Y., Stachelberger H., Brzezinski M.A., Stucky G.D., Morse D.E., Hansma P.K. (2003). Atomic force microscopy study of living diatoms in ambient conditions. J. Microsc..

[29] Majewska R., Gambi M.C., Totti C.M., Pennesi C., Stefano M. (2012). Growth form analysis of epiphytic diatom communities of Terra Nova Bay (Ross Sea, Antarctica). Polar Boil..

[30] Donev A., Connelly R., Stillinger F.H., Torquato S. (2007). Underconstrained jammed packings of nonspherical hard particles: Ellipses and ellipsoids. Phys. Rev. E Stat. Nonlinear Soft Matter Phys..

